# Selenate Reduction and Selenium Enrichment of Tea by the Endophytic *Herbaspirillum* sp. Strain WT00C

**DOI:** 10.1007/s00284-019-01682-z

**Published:** 2019-04-08

**Authors:** Xiao Xu, Wei Cheng, Xin Liu, Heng You, Guitai Wu, Kunming Ding, Xiuliang Tu, Lanfang Yang, Youpin Wang, Yadong Li, Haoshuang Gu, Xingguo Wang

**Affiliations:** 1 The Faculty of Life Science, Hubei University, Wuhan, China; 2 The Faculty of Physics and Electronic Sciences, Hubei University, Wuhan, China; 3 Xianning Academy of Agricultural Science, Xianning, Hubei China; 4 The Faculty of Resource and Environmental Science, Hubei University, Wuhan, China; 5 Hubei Academy of Agricultural Science, Wuhan, China

## Abstract

*Herbaspirillum* sp. WT00C is a tea-plant-specific endophytic bacterium. A genomic survey revealed an intact pathway for selenocompound metabolism in the genome of this bacterium. When it was cultured with sodium selenate, *Herbaspirillum* sp. WT00C was able to turn the culture medium to red. Electron microscopy and energy-dispersive X-ray spectroscopy confirmed that *Herbaspirillum* sp. WT00C reduced selenite (Se^6+^) to elemental selenium (Se^0^), and selenium nanoparticles (SeNPs) were secreted outside bacterial cells and grew increasingly larger to form Se-nanospheres and finally crystallized to form selenoflowers. Biochemical assays showed that selenospheres contained proteins but not carbohydrates or lipids. The improvement of selenium enrichment of tea plants by *Herbaspirillum* sp. WT00C was also tested. After *Herbaspirillum* sp. WT00C was inoculated into tea seedlings via needle injection and soaking tea-cutting methods, this endophytic bacterium markedly enhanced selenium enrichment of tea. When the tea seedlings inoculated by soaking tea-cutting mode were cultivated in the selenium-containing soils, selenium contents of tea leaves in three experimental groups were more than twofold compared to those of control groups. Our study demonstrates that the endophytic bacterium *Herbaspirillum* sp. WT00C has the ability to reduce selenate and improve selenium enrichment of tea.

## Introduction

Selenium is well known as an essential trace element for both plants and mammals. Typically, selenium exists as selenate and selenite oxoanions in soil. Plants can absorb selenium directly from soil via roots. Low doses of selenium can stimulate the growth of plants, whereas at high dosages, it can cause plant damage [1–3]. Mammals only obtain selenium indirectly through food. In both plants and mammals, selenium compounds are typically metabolized into selenocysteine and selenomethionine. The deficiency of selenium is thought to be associated with over 40 human diseases [4, 5], and selenium has also been shown to be effective against cancers [6, 7].

Many bacterial species have been observed to reduce selenate/selenite to red elemental selenium [8–13]. The detoxification of selenates/selenites to nontoxic elemental selenium was catalyzed by microbes, and the reverse process occurs slowly. This redox process is both time and concentration dependent [14]. Different bacterial strains reduce selenate/selenite to selenium nanoparticles under different conditions, including aerobic and anaerobic conditions [15–18]. Different types of selenium nanoparticles have been synthesized using proteins, peptides, and several other reducing agents [11, 14, 19]. However, a detailed mechanism for the formation and transformation of selenium nanospheres has not been fully elucidated.

*Camellia sinensis* (L.) is native to East, South, and Southeast Asia, and is currently cultivated in tropical and subtropical regions worldwide. Epidemiological observations and laboratory studies indicate that polyphenolic compounds present in tea may reduce the risk of a variety of illnesses, including cancer and coronary heart disease [20]. Animal model studies show that tea consumption protects against lung, forestomach, esophagus, duodenum, pancreas, liver, breast, colon, and skin cancers induced by chemical carcinogens [21–25]. Green tea consumption also has preventive effects against atherosclerosis and coronary heart disease, high blood cholesterol, and high blood pressure [26–28]. Selenium is an essential trace element that has a close relationship with cancers, hypertension, metabolic syndrome, diabetes, and other types of diseases due to its biological functions, such as in antioxidation, thyroid metabolism, protein folding, redox signaling, and immune system activation [29]. Thus, selenium-rich tea has received increased attention recently. However, most lands have selenium-poor soil, and the selenium content in agronomic crops is quite low (< 0.1 mg/kg). Only in selenium-rich areas or in tea gardens that have applied selenite fertilizer or selenite directly via foliage sprays, the selenium content in tea leaves approach more than 0.25 mg/kg. Even in the so-called high-selenium-enriched tea, the selenium content is only approximately 5 mg/kg, which is too low for using as dietary supplement in humans. How to further improve the selenium content of tea remains an unsolved issue.

*Herbaspirillum* sp. WT00C, which was isolated from *Camellia sinensis* L and classified as a novel member of the genus *Herbaspirillum*, was able to grow slowly in nutrient broth (NB) or Luria–Bertani (LB) medium under laboratory conditions [30]. This bacterium entered into plants via traumatic infection and colonized only in tea plants without any disease symptoms [31]. Unlike *H. seropedicae*, *H. rubrisubalbicans*, and *H. lusitanum* which were able to fix nitrogen [32–34], *Herbaspirillum* sp. WT00C did not exhibit nitrogen-fixing activity, and did not colonize in other crops, such as *Brassica campestris*, *Brassica rapa*, *Oryza sativa*, and *Triticumaestivum* [31]. Similar to *H. seropedicae*, *H. rubrisubalbicans,* and *H. lusitanum,* this tea-specific endophytic bacterium was also observed to produce indole-3-acetic acid (IAA), ammonia and siderophores [30, 31], which was thought to play a role in inducing adventitious formation in dicots or monocots and improving plant growth and development [32–36]. Laboratory and field studies showed *Herbaspirillum* sp. WT00C indeed promoted lateral root formation and bud growth of tea cuttings and improved the growth of tea plants in field experiments. Thus, it was suggested that *Herbaspirillum* sp. WT00C could be developed as a plant growth-promoting agent used specifically for tea cultivation [31]. The genome of *Herbaspirillum* sp. WT00C was sequenced [37] and deposited in GenBank (Acc#: MIJG00000000). In this study, we identified a pathway for selenocompound metabolism through investigation of its genome, in which selenate and selenite can be reduced to zerovalent selenium or incorporated into proteins through selenocysteine or selenomethionine. Next, we examined the process of selenate reduction in detail and confirmed that this bacterium indeed reduced selenate to elemental selenium and produced selenium nanospheres outside of bacterial cells. We hypothesized that the selenium reduction and metabolic capabilities of *Herbaspirillum* sp. WT00C might improve the selenium enrichment of tea when the bacterium colonized inside tea plants. To test this hypothesis, we inoculated the bacterium into tea plants via either needle injection or soaking tea cuttings. The results obtained from both methods provided convincing evidence that *Herbaspirillum* sp. WT00C significantly improved the selenium enrichment of tea. Meanwhile, a plausible mechanism for this observation was also suggested.

## Materials and Methods

### Bacterial Strain and Culture

*Herbaspirillum* sp. WT00C, an endophytic bacterium, was isolated by our laboratory from an ornamental tea plant (*Camellia sinensis* L.) in the moshan scenic spot of Wuhan city and classified as a novel member in the genus *Herbaspirillum* [30]. A brief description of bacterial isolation and identification is given below, the conventional endophyte isolation was performed by clearing the tea-plant samples with water, sterilizing with 75% alcohol for 1 min and then 1% mercury bichloride for 3 min, and finally washing 3–5 times with sterilized dH_2_O. Then the sterilized sample was cut into 3 × 3 cm tissue blocks, and cultured on nutrient agar (NA) containing 0.3% beef extract, 1% peptone, 0.5% NaCl, 1.5% agar, with pH 7.5 at 28 °C for 48 h. The organisms isolated from *Camellia sinensis* L. were diluted and spread on the NA plates, and cultivated at 28 °C for 48 h. All colonies obtained from the plates were transferred into fresh NA plates and incubated for another 48 h at 28 °C, in which a round, opaque, milky white, surface-raised, neat-edge colony was selected. Subcultivation was performed in nutrient broth (NB, 0.3% beef extract, 1% peptone, 0.5% NaCl, pH 7.5) at 28 °C for 48 h, and the isolated strain was named as the strain WT00C [30]. Its 16S rRNA gene sequence (MK119980) shared 98–99% homologous similarity with those strains in *Herbaspirillum* genus, and phylogenetic analysis also showed that the strain WT00C was affiliated with the genus *Herbaspirillum* [30]. The strain WT00C was Gram-negative, micro-aerobic, rod-shaped, 1.4–1.8 μm in length and 0.5–0.7 μm in width, polar flagellum, and nonpigmented. Colonies growing on NB or Luria–Bertani (LB) agar were round, convex, opaque, milky white, surface raised, and had neat edges. Growth occurred up to 40 °C in pH 5.0–8.0 and 0–1.5% (w/v) NaCl. Cells, producing indoleacetic acid (IAA), ammonia, and siderophore, were weakly resistant against 10 μg/mL penicillin and 5 μg/mL spectinomycin, but susceptible to chloramphenicol, tetracycline, gentamicin, kanamycin, erythromycin, and streptomycin [30]. The strain WT00C, detected by APIZYM25200 (BioMérieux) according to the manufacturer’s instruction, also displayed enzymatic activities of alkaline phosphatase, esterase (C4), esterase lipase (C8), leucine arylamidase, valine arylamidase, acid phosphatase, and naphthol-AS-BI-phosphohydrolase.

This bacterium was routinely cultured in NB or LB medium. At first, the strain WT00C was picked from − 80 °C stock culture and streak inoculated on the NA plate containing 5 μg/mL spectinomycin and 10 μg/mL ampicillin, and then incubated at 37 °C overnight. Single colony was inoculated into 5 mL NB medium supplemented with 5 μg/mL spectinomycin and 10 μg/mL ampicillin. After incubation at 37 °C overnight, the culture was transferred into fresh LB medium at an inoculation ratio of 1:100 and grown at 37 °C as described previously [30, 31].

### Tests of Selenate Reduction

Three methods were used to test the selenate reduction of *Herbaspirillum* sp. WT00C. Before the test of selenate reduction, the strain WT00C was first picked from − 80 °C store culture and streak-inoculated on the NA plate containing 5 μg/mL spectinomycin and 10 μg/mL ampicillin, and then incubated at 37 °C overnight. Single colony was picked and inoculated into 5 mL NB broth supplemented with 5 μg/mL spectinomycin and ampicillin, and then incubated at 37 °C overnight for bacterial activation. Second, three methods were used to examine if the activated bacterial cells were the strain WT00C: (1) The bacterial cells were stained with Gram-staining kit (Sigma-Aldrich) and then cell size, morphology, and Gram-staining were observed under a microscope (Dianying, DYS-106); (2) IAA production and antibiotic susceptibility were tested as described previously [30, 31]; and (3) The enzymatic profile was examined by API ZYM25200 (BioMérieux) according to the manufacturer’s instruction. To test the selenate reduction, the bacterial culture was spread on six LB plates containing 5 μg/mL spectinomycin, 10 μg/mL ampicillin, and 0 or 25 mM sodium selenate after identification of the activated bacterial cells. Three LB plates without addition of sodium selenate were used as control. After incubation at 37 °C for 24 h, the color of bacterial colonies was observed and recorded by photography. Meanwhile, color change was also observed in LB broth. The activated bacterial cells were inoculated with the ratio of 1:100 into six flasks containing 200 mL fresh LB medium containing 5 μg/mL spectinomycin and 10 μg/mL ampicillin, in which three flasks were supplemented with 200 mM sodium selenate and another set of 3 flasks without addition of sodium selenate, which were used as control. All flasks were incubated at 37 °C, at 200 rpm for 24 h. The color of bacterial cultures was observed and recorded by photography.

Selenate depletion and selenite formation were examined by means of Hydride-generation atomic fluorescence spectrometer (AFS200, Skyray Instrument Inc.). The overnight-activated bacterial cells were inoculated into 10 mL LB broth supplemented with 5 μg/mL spectinomycin and 10 μg/mL ampicillin, and then incubated at 37 °C until OD_600_ reached 0.8. Then, 0.1 mL of the bacterial culture was transferred to 10 mL LB medium, containing 5 μg/mL spectinomycin, 10 μg/mL ampicillin, and 1 mg/mL sodium selenate, and incubated at 37 °C, at 200 rpm for 0, 2, 5, 10, and 15 h. Bacterial cultures were, respectively, centrifuged at 6000×*g*, 4 °C for 15 min. After centrifugation, the pellet was removed away, and only the supernatant was collected. The supernatants collected at different incubation times were divided into two parts: one was used for the determination of total selenium content, and the other for selenite determination. Sample preparation and selenium measurement were performed according to the National standards of the People’s Republic of China (GB 5009.93). In brief, 10 ml of the supernatant for each sample was poured into a beakerflask, and heated on a graphite heating plate at 180 °C until 2 mL residual solution left. After the flask was cooled, 10 mL of the mixed acid solution (1 nitric acid: 4 perchloric acid) was added, mixed, and allowed to stand at room temperature overnight, and then the flask was heated to 180 °C again. When the flask was cooled, 10 ml 6 M HCl was added, and then the standing and heating processes described above were executed again. The solution in the flask was transferred to a 25-mL volumetric flask, and the capacity was made up to 25 mL with ultrapure water. 5 mL solution was taken and transferred to another 25-mL volumetric flask, where 1 mL 10 mg/mL potassium ferricyanide and 2 mL 18% HCl were added and then the capacity made up to 25 mL. Those samples prepared above were used for determining total selenium content. The procedure of sample preparation for selenite (SeO_3_^2−^) determination was similar to that for total selenium measurement except for two steps of acid treatments with boiling. Each sample, the carrier (4% HCl), and the reducer (0.5% potassium hydrate and 1.5% potassium borohydride) were, respectively, loaded into AFS200 via autoloaders, and measurements and data collections were performed according to the manufacturer’s instruction. Measurement for each sample was repeated three times. Selenium content was finally calculated based on the standard curve which was prepared by the selenium standard purchased from Tanmo standard substance Center (Beijing). Selenate residue content was calculated based on the Eq. 1.

1 $${\text{Selenate residue }}\left( {{\text{mg}}/{\text{ml}}} \right) \, = {\text{ the}\, \text{total}\, \text{selenium}\, \text{measured}}\left( {{\text{mg}}/{\text{ml}}} \right) \, {-}{\text{ the} \,\text{selenite}\, \text{measured}}\left( {{\text{mg}}/{\text{ml}}} \right)$$

### Transmission Electron Microscopy (TEM)

*Herbaspirillum* sp. WT00C was grown in 25 mL LB broth containing 200 mM selenate at 37 °C for 0, 5, 7, 10, 15, 20, and 24 h. The bacterial cultures were then diluted 20-folds with phosphate buffer (200 mM, pH 7.2). Next, the diluted cultures were aliquoted onto a formvar carbon support film, which was incubated at room temperature for several hours to allow for water evaporation. Finally, the samples were observed under a Tecnai G^2^ 20 transmission electron microscope (FEI, USA) at 200 kV.

### Scanning Electron Microscopy (SEM)

SEM was used to observe bacterial cells and SeNPs as reported by Li et al. [38]. In brief, *Herbaspirillum* sp. WT00C was grown in 25 mL LB broth containing 200 mM selenate at 37 °C for 12, 24, or 48 h. The bacterial cultures were centrifuged at 6000×*g* for 15 min, and the pellets were fixed in 2.5% glutaraldehyde for 30 min, rinsed 3 times in 100 mM phosphate buffer (pH 7.2), and dehydrated in an ethanol series (50, 70, 90, and 100% ethanol). The ethanol was then displaced by isoamyl acetate, and the samples were mounted onto microscope slides and dried using a BAL-TEC CPD030 critical point drying apparatus. Finally, the samples were sputter-coated with gold to a thickness of approximately 20 nm and observed under a JSM7100F scanning electron microscope (JEOF, Tokyo, Japan).

### X-ray Diffraction Analysis

*Herbaspirillum* sp. WT00C was cultured in 50 mL LB broth containing 50 or 200 mM selenate at 37 °C for 24 h, after which the cultures were centrifuged at 6000×*g* and 4 °C for 20 min. The supernatants were discarded, and the pellets containing bacterial cells and SeNPs were resuspended in 50 mM Tris–HCl (pH 7.5) and then centrifuged again, with this washing step repeated twice. The pellets were collected, freeze-dried in a LGJ-10 vacuum freeze dryer (Songyuan Beijing), and ground to fine particles with a diameter of 40 μm. Finally, the powdered samples were analyzed using a D8A25 X-ray diffractometer (Bruker, Germany) using Cu Kα radiation (*λ *= 1.5406 Å) in the range of 5° to 80° at 40 kV.

### Selenate Reduction in Cytoplasmic and Cell-Debris Protein Fractions

*Herbaspirillum* sp. WT00C was cultured in LB broth at 37 °C for 15 h. When the OD_600_ value approached to 1.0, the cells were harvested by centrifugation at 800×*g*, 4 °C for 15 min. Next, the cells were resuspended in PBS (pH 7.2) and washed and centrifuged at 4 °C three times with the same buffer to remove the entire LB medium. The bacterial cells in PBS (pH 7.2) were sonicated, and the supernatant and the pellet were separated by centrifuging at 10,000×*g*, 4 °C for 20 min. The cytoplasmic protein-containing supernatant was collected, and the membrane protein-containing pellet was dissolved in PBS (pH 7.2). Protein concentrations were determined using the Bradford method [39]. Equal amount of proteins (0.3 mg/mL) were used to assess selenate reduction activity. Reactions in 1 mL volume were initiated by the addition of Na_2_SeO_4_ to a final concentration of 50 mM at 37 °C for 0–34 h. Selenate reduction was observed by eye at different times based on the formation of a red-color appearance.

### Purification and Assay of Selenium Nanospheres

*Herbaspirillum* sp. WT00C was cultured in LB broth containing 200 mM Na_2_SeO_4_ at 37 °C for 36 h and then centrifuged at 800×*g*, 4 °C to remove bacterial cells. The supernatant was collected and centrifuged again at 6080×*g*, 4 °C to remove all liquid. The red precipitate was resuspended in PBS (pH 7.2) and then centrifuged at 6080×*g*, 4 °C, and this step was repeated three times. Finally, the red precipitate was resuspended in PBS. The Se-nanospheres were further purified by performing a 50–70% sucrose density gradient centrifugation at 12,000×*g*, 4 °C for 30 minus as described by Dobias et al. [40]. The red Se-nanosphere band was carefully removed and washed twice with PBS by means of centrifugation at 6080×*g*, 4 °C for 20 min to remove all sucrose. The purified Se-nanospheres were then suspended in PBS.

Fehling’s reagent was used to test for reducing and nonreducing sugars referring to the Chinese national standard (GB 5513-85), Sudan II [1-(2,4-xylidylazo)-2-naphthol] was used to test the lipoid component, the TLC method [41] was used to test phospholipids, and 10% SDS-PAGE and Coomassie blue staining were used to test for the presence of proteins in Se-nanospheres.

### Bacterial Inoculation and Tea-Cutting Propagation

*Camellia sinensis* cv. Chuyeqi was obtained from the Xianning Academy of Agriculture Science and used as parent materials in this study. Tea-cutting propagation was performed according to a conventional approach [31]. In brief, tender shoots of the tea plant in the tea farm were used to prepare tea cuttings with lengths of 5–7 cm. Each cutting carried a single node and a leaf at its upper part. Tea cuttings were planted into seedbeds and cultivated into seedlings.

Two strategies were used for bacterial inoculation. A needle injection method was used to test whether *Herbaspirillum* sp. WT00C improved selenium enrichment of tea, and tea cuttings were inoculated by soaking to develop a technique for field application. In the needle injection experiment, two-year-old tea seedlings in tea garden were transplanted into pots containing 10 kg of soil and were cultivated in a greenhouse. Experiments were performed in four groups. The first group was used as control. In the other three groups, Na_2_SeO_4_ was used to spike the soil, and then the selenium contents of soils were assayed. Final selenium contents of soils in four groups were 0, 0.622, 1.5, and 3.5 mg/kg. In each group, 100 tea seedlings were cultivated. When tea seedlings sent out young sprouts with 2–3 leaves, equal amounts of bacterial cells (3 × 10^10^ cfu) were injected into the tea stems at 5 cm above the roots using a sterile needle, after which the wounds were covered by a piece of cotton wool. In the control groups, an equal volume of fresh LB medium was injected. After injection, all seedlings were continually cultivated in a greenhouse.

Soaking inoculation of tea cuttings was performed based on a method that was reported by our group [31]. In brief, the bacterial culture was prepared as described above, and then glycerol was added to a final concentration of 5%. After the culture was further diluted with H_2_O (2 bacterial culture:1 H_2_O), tea cuttings were soaked in the diluted culture for 1 h. In the control group, tea cuttings were soaked in water under the same conditions. After treatment, all tea cuttings were removed from the liquid and dried until no liquid dripping was observed. Next, tea cuttings were immediately planted in a nursery, and field management was performed according to the routine methods used for tea-cutting propagation. After 1 year, tea seedlings were transplanted into pots containing 10 kg of soil with selenium contents of 0, 0.622, 1.5, or 3.5 mg/kg and were cultivated in natural environments. Each of the four groups contained 100 tea seedlings.

### Sample Collection and Determination of Selenium Content

After treatment, 25 tea seedlings in each group were taken out after 30, 60, 90, and 150 days and were washed three times with water. The roots, stems, and leaves in each group were separately collected, dehydrated to dryness in a 70 °C oven, and then ground to a powder. After the powder samples were passed through a 100-mesh screen, the 100-mesh powder of each sample was collected in a sample bag. The selenium content was finally measured using the atomic fluorescence spectrometer (AFS200) according to the National standards of the People’s Republic of China (GB/T 21729). Data were collected and analyzed using SPSS24.0, and a *P* value of less than 0.05 was considered significant.

## Results

### Selenate Reduction of *Herbaspirillum* sp. WT00C

**Fig. 1:**
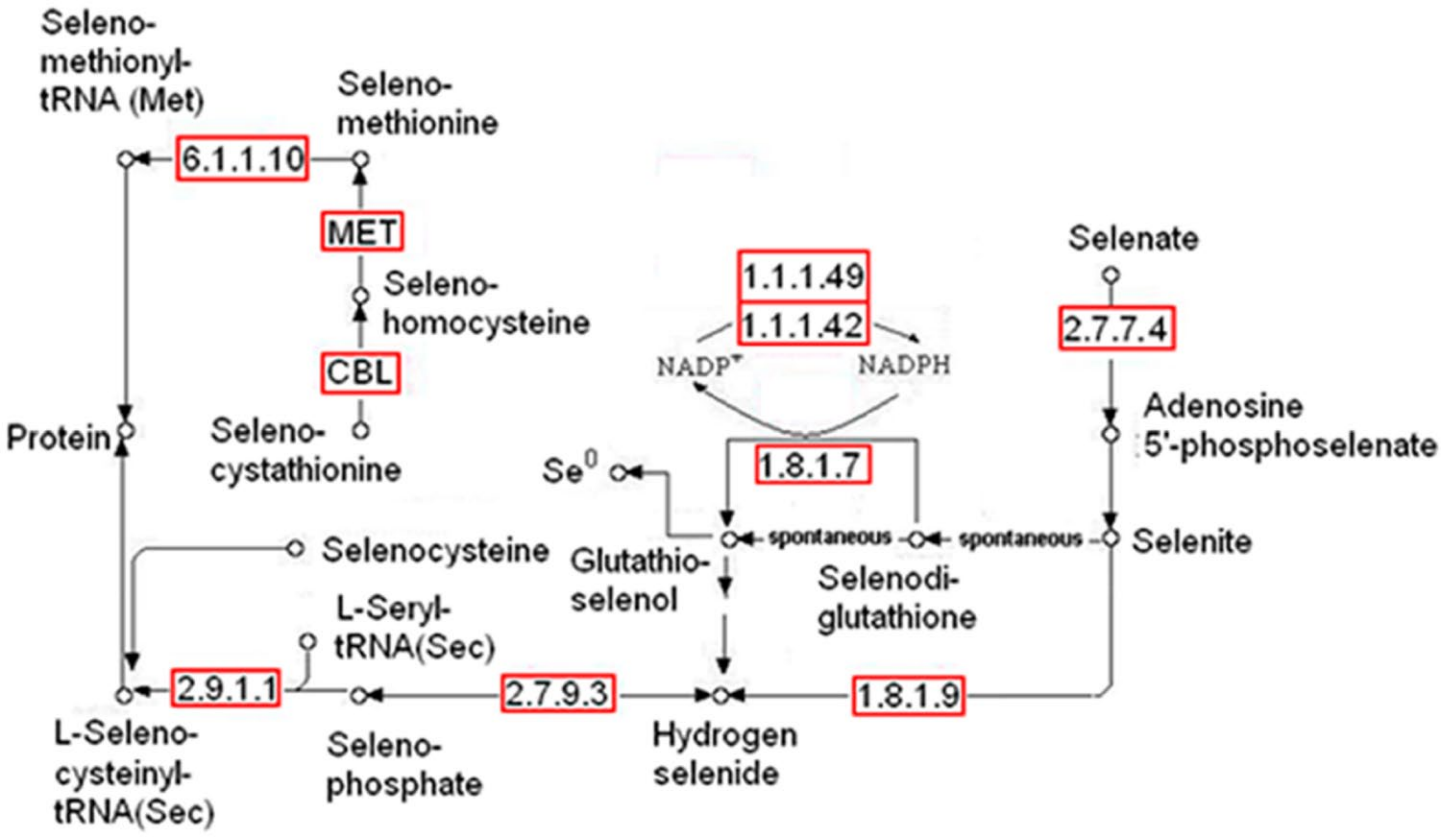
The pathway of selenocompound metabolism in *Herbaspirillum* sp. WT00C

**Fig. 2:**
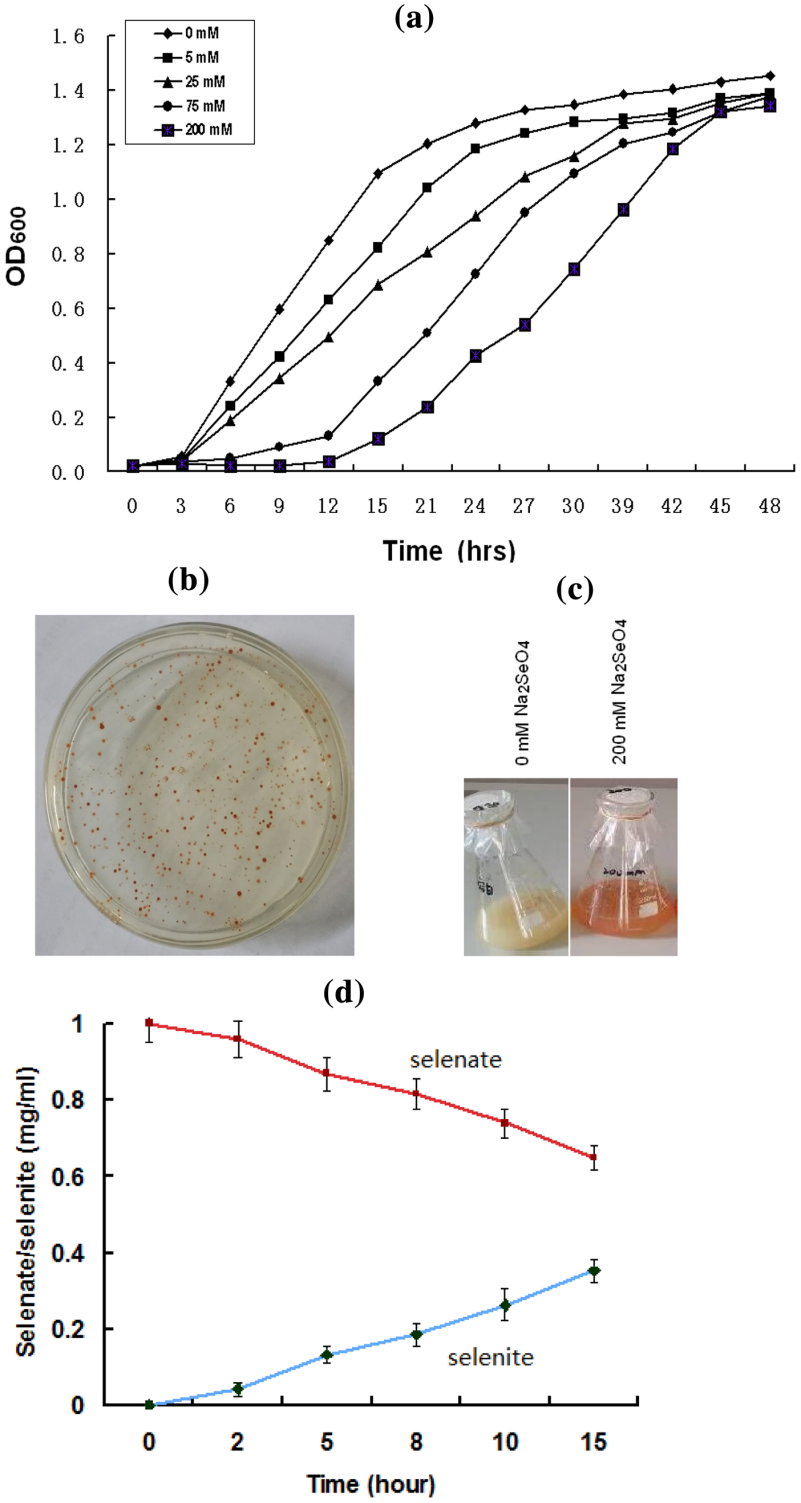
Growth inhibition and selenate reduction of *Herbaspirillum* sp. WT00C. **a** Growth curves of *Herbaspirillum* sp. WT00C in LB broth with different selenate concentrations (0–200 mM); **b** Colonies of *Herbaspirillum* sp. WT00C growing at 37 °C on the LB plate containing 25 mM selenate; **c** Selenate reduction by *Herbaspirillum* sp. WT00C in 200 mL LB medium containing 0 or 200 mM Na_2_SeO_4_ at 37 °C for 24 h; **d** Selenate depletion and selenite formation curves of *Herbaspirillum* sp. WT00C incubating in the LB medium containing 1 mg/mL sodium selenate at 37 °C for 0–15 h

As reported previously [37], the genomic survey of *Herbaspirillum* sp. WT00C by means of the analysis technique of bioinformatics resulted in the identification of an intact selenocompound metabolic pathway. In this pathway, selenate is initially converted to selenite, and then selenite is reduced to zerovalent elemental selenium via two intermediates, namely, selenodiglutathione and glutathioselenol, or incorporated into proteins through selenocysteine (see Fig. 1). This bacterium was also found to have an intact pathway for glutathione synthesis. Glutathione was thought to play an important role in selenite reduction [42]. To test if *Herbaspirillum* sp. WT00C reduces selenate to elemental selenium, it was initially grown at 37 °C on an LB plate containing 25 mM sodium selenate, and all colonies became red in appearance (Fig. 2b). In contrast, the color of bacterial colonies in the control was unchanged (data not shown). Next, *Herbaspirillum* sp. WT00C was also inoculated into LB broth containing 0 and 200 mM sodium selenate and grown at 37 °C for 24 h. As shown in Fig. 2c, the medium color became red, whereas the color of the control without sodium selenate did not change. Finally, selenate depletion and selenite formation by *Herbaspirillum* sp. WT00C were also detected. As shown in Fig. 2d, selenate was gradually depleted when it was incubated in the LB medium containing 1 mg/mL sodium selenate at 37 °C for 0–15 h, and selenate residue at 15 h was approximately 75%. Meanwhile, selenite was gradually increased under the same condition. Red color appetence, selenate depletion, and selenite formation indicated that this bacterium was able to reduce selenate to selenite and then form red elemental selenium (Se^0^).

The growth of *Herbaspirillum* sp. WT00C in LB broth with different selenate concentrations (0–200 mM) was investigated, and the growth curve is shown in Fig. 2a. When a high concentration (200 mM) of Na_2_SeO_4_ was used, bacterial growth was observably inhibited during 12-h cultivation. After 12 h, bacterial cells gradually recovered their growth, and finally approached to the wild-type level after 48 h (see Fig. 2a). This result suggested that this bacterium appears to have a strong selenate tolerance. Viable bacterial count on LB plates showed that bacterial cells were viable when 200 mM selenate was used. The numbers of viable cells observed at 0, 12, 24, and 48 h were 2 × 10^6^, 3.3 × 10^6^, 4.2 × 10^8^, and 1.4 × 10^11^ cfu/mL, respectively, demonstrating that bacterial cells kept alive during 12-h incubation and recovered their growth after a 12-h inhibition.

**Fig. 3:**
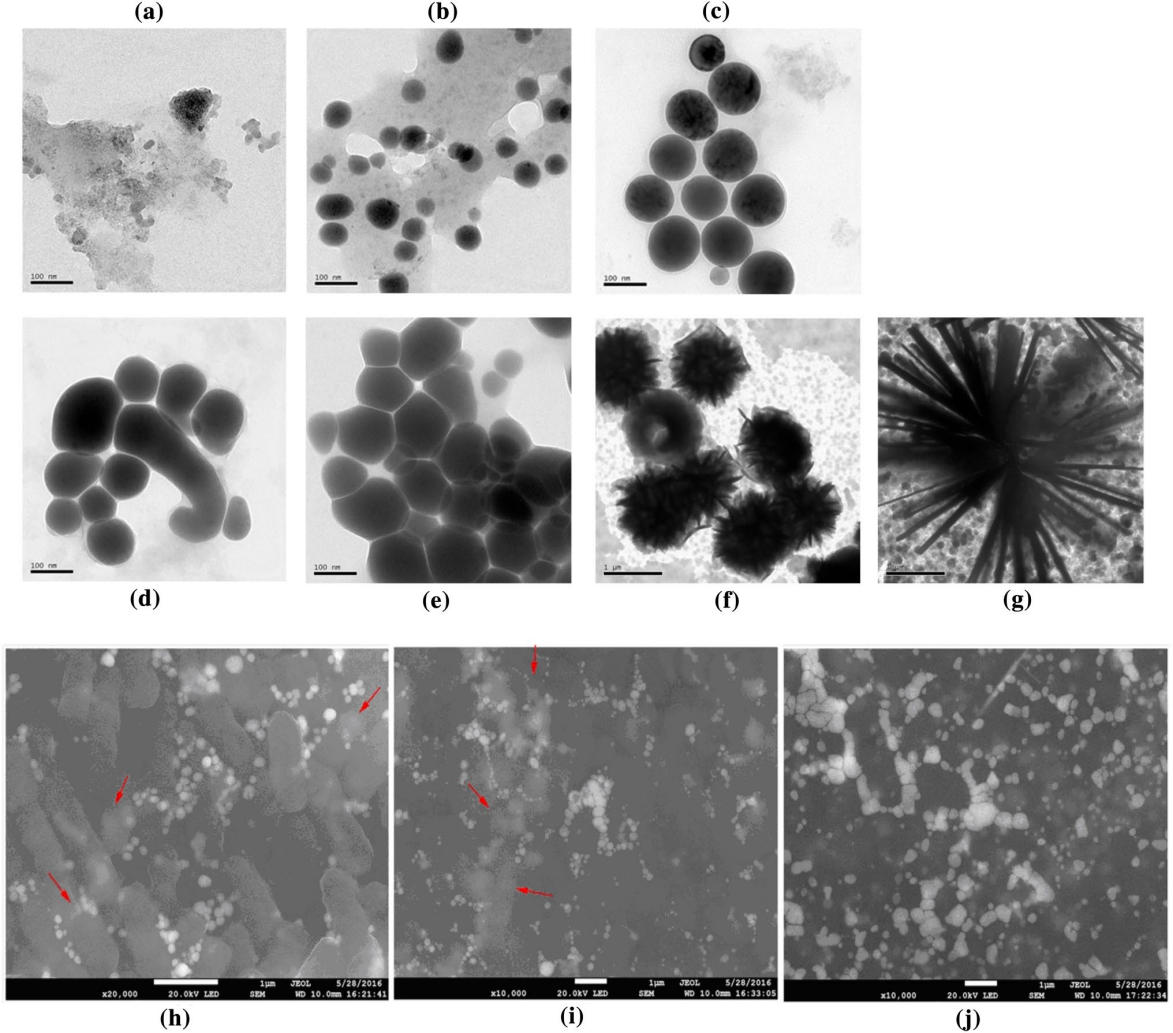
TEM and SEM observations of selenium nanoparticles. **a**–**g** TEM observations of selenium nanoparticles at 5, 7, 10, 15, 20, and 24 h of bacterial culturing at 37 °C with 200 mM NaSeO_4_. **h**–**j** SEM observations of bacterial cells and selenium nanoparticles at 12, 24, and 48 h of bacterial culturing with 200 mM NaSeO_4_ at 37 °C. SeNPs scattered as dispersed shapes on cell surfaces were indicated by a red arrowhead

*Herbaspirillum* sp. WT00C was grown in LB broth containing 200 mM Na_2_SeO_4_ at 37 °C for 0–24 h, and culture samples were taken at 5, 7, 10, 15, 20, and 24 h and observed by transmission electron microscopy (TEM). TEM revealed that *Herbaspirillum* sp. WT00C reduced selenate (Se^6+^) to elemental selenium (Se^0^) nanoparticles (SeNPs), which ranged in size from a few nanometers to more than 200 nm (Fig. 3). As shown in Fig. 3, SeNPs were spherical in shape and gradually increased in size as the bacterial incubation time increased. Selenium nanoparticles initially gathered together to form selenium nanospheres outside bacterial cells (Fig. 3a, b) and then grew larger through fusion between small Se-nanospheres (Fig. 3d, e), and finally crystallized to form selenium flowers with diameters of more than 1 μm (Fig. 3f, g). The results from scanning electron microscopy (SEM) observation gave more detailed information regarding bacterial cells and SeNPs. As shown in Fig. 3h–j, bacterial cells were intact, and SeNPs initially assembled onto cell surfaces as dispersed shapes and then formed Se-nanospheres outside bacterial cells. The SeNPs scattered as a dispersed shape on the cell surfaces suggested that they could be secreted from the bacterial cytoplasm to the exterior of cells, where Se-nanospheres were subsequently formed.

**Fig. 4:**
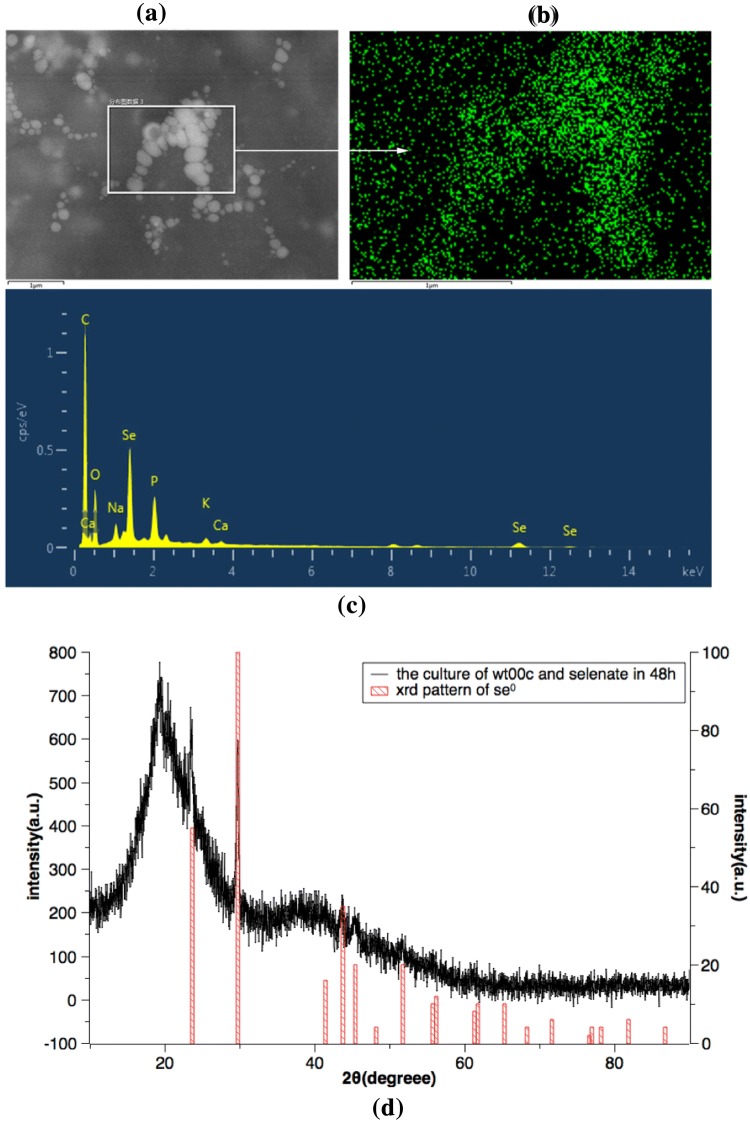
EDS and XRD analyses of spectrum patterns. **a** SEM observation of bacterial cells and Se-nanospheres; **b** Se-nanospheres displayed a strong blue selenium spectrum; **c** Electromagnetic emission spectra of chemical elements of bacterial cells and SeNPs in which selenium occurred as a large peak; **d** XRD spectral patterns of bacterial cells and SeNPs, which matched perfectly with the standard spectrum of elemental selenium (Se^0^)

To further confirm whether *Herbaspirillum* sp. WT00C reduced selenate to elemental selenium (Se^0^), a bacterial culture sample at 24 h described above was analyzed using energy-dispersive X-ray spectroscopy (EDS). As shown in Fig. 4, the Se-nanospheres gathered together in the box showed a strong blue spectrum of selenium (Fig. 4b) and a typical peak for the electromagnetic emission spectrum of selenium (Fig. 4c). X-ray diffraction (XRD) patterns also showed that this mixture containing bacterial cells and the extracellular red SeNPs had four intense peaks over the whole spectrum of the 2 values ranging from 5 to 90. These intense peaks matched perfectly with the standard spectrum of elemental selenium (Se^0^); obtained from the database of the National Research Council of Canada Crystallographic Data File, NRCC) but did not match with the standard spectrum of Se^6+^ (data not shown). These results clearly confirmed that *Herbaspirillum* sp. WT00C reduced selenate (Se^6+^) to elemental selenium (Se^0^).

**Fig. 5:**
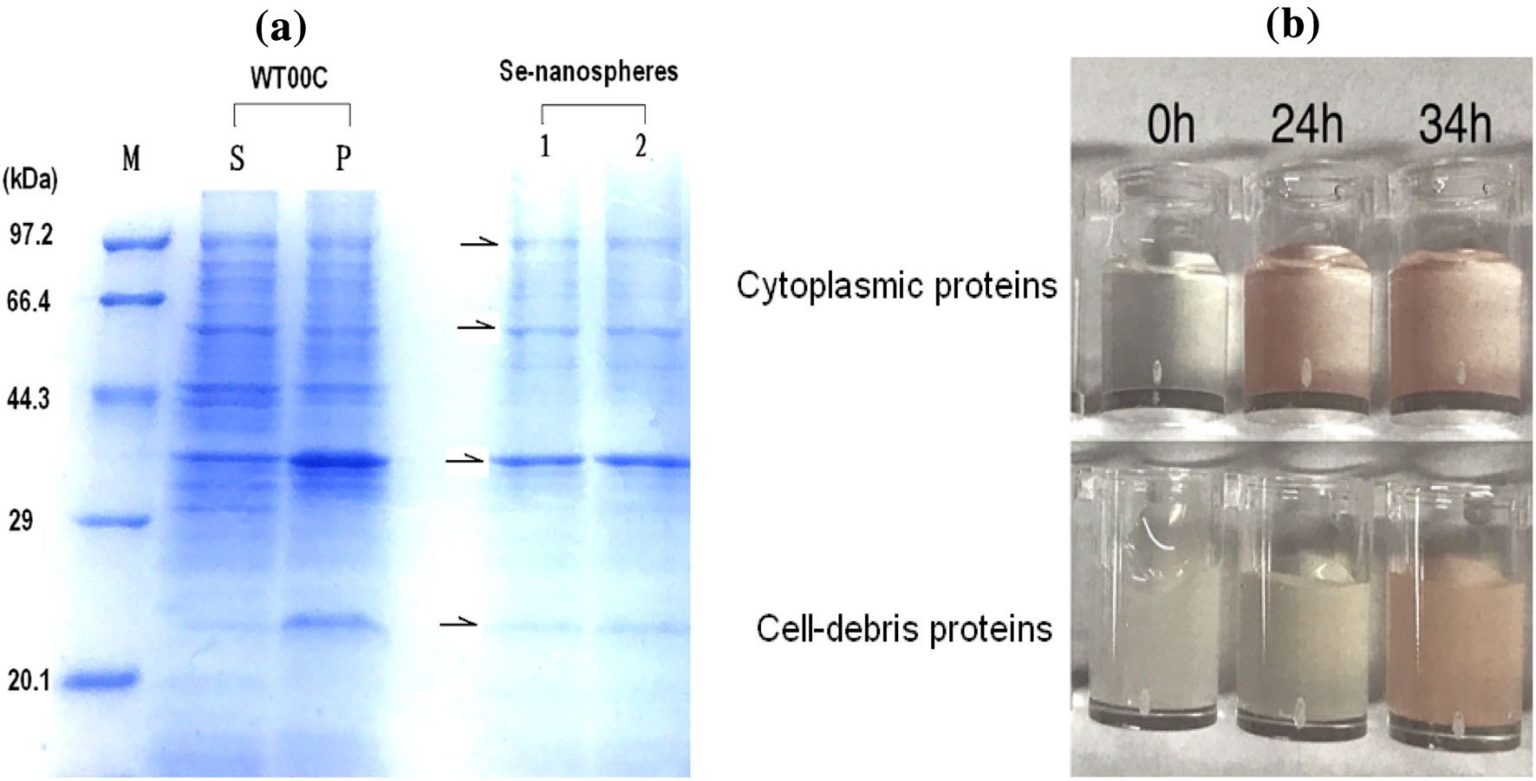
Protein assay of Se-nanospheres and activity test of selenate reduction of cytoplasmic and cell-debris proteins. **a** Protein assay of Se-nanospheres by 10% SDS-PAGE. The primary protein band is indicated by pointed by an arrow. WT00C: *Herbaspirillum* sp. WT00C; S: supernatant; P: pellet; M: protein marker. **b** Selenate reduction activities of cytoplasmic and cell-debris proteins. In both cases, 50 mM Na_2_SeO_4_ and 0.3 mg/mL protein were mixed in 50 mM phosphate buffer (pH 7.2) and then incubated at 37 °C for different times

To test the selenate reduction activities of cytoplasmic proteins and cell debris, 50 mM Na_2_SeO_4_ was added to 0.3 mg/mL proteins, and the selenate reduction activity was observed at different times. As shown in Fig. 5b, cytoplasmic proteins displayed a red color after 24 h, and the proteins from cell debris produced a red color after 34 h under the same conditions, indicating that the activity of selenate reduction of cytoplasmic proteins was more active than that of bacterial membranes. This observation suggested that the reaction of selenate reduction occurs inside and outside bacterial cells but predominantly occurs inside cells.

Se-nanospheres were purified from bacterial cultures and tested with Fehling’s reagent, but both reducing and nonreducing sugars were undetectable. The Sudan II test also gave negative result, suggesting that Se-nanospheres did not contain lipoid. In addition, the results of a TLC analysis did not identify any phospholipids in Se-nanospheres. In contrast, a 10% SDS-PAGE gel showed that Se-nanospheres contained proteins, which exhibited four major protein bands with molecular weights of 92, 56, 37, and 23 kDa (see Fig. 5). These results indicated that Se-nanospheres contain proteins but not carbohydrates or lipids. As shown in Fig. 5, the proteins of Se-nanospheres appeared to come from bacterial cells.

### Improvement of Selenium Enrichment of Tea by *Herbaspirillum* sp. WT00C

*Herbaspirillum* sp. WT00C is a gram-negative, tea-specific endophytic bacterium [30, 31] that enters tea plants via traumatic infection and primarily colonizes inside stems or old leaves of tea plants [33]. Because of its ability to reduce selenate to form protein-containing Se-nanospheres, this bacterium might improve selenium enrichment of tea when it colonizes tea plants growing in selenate-containing soils. To test this possibility, we designed two methods, needle injection and soaking tea cuttings, to inoculate *Herbaspirillum* sp. WT00C into tea plants. The two methods were assessed because the needle injection method is only used for laboratory test but is not applicable for use in tea fields, whereas the soaking tea cutting method can be used in tea fields.

**Fig. 6:**
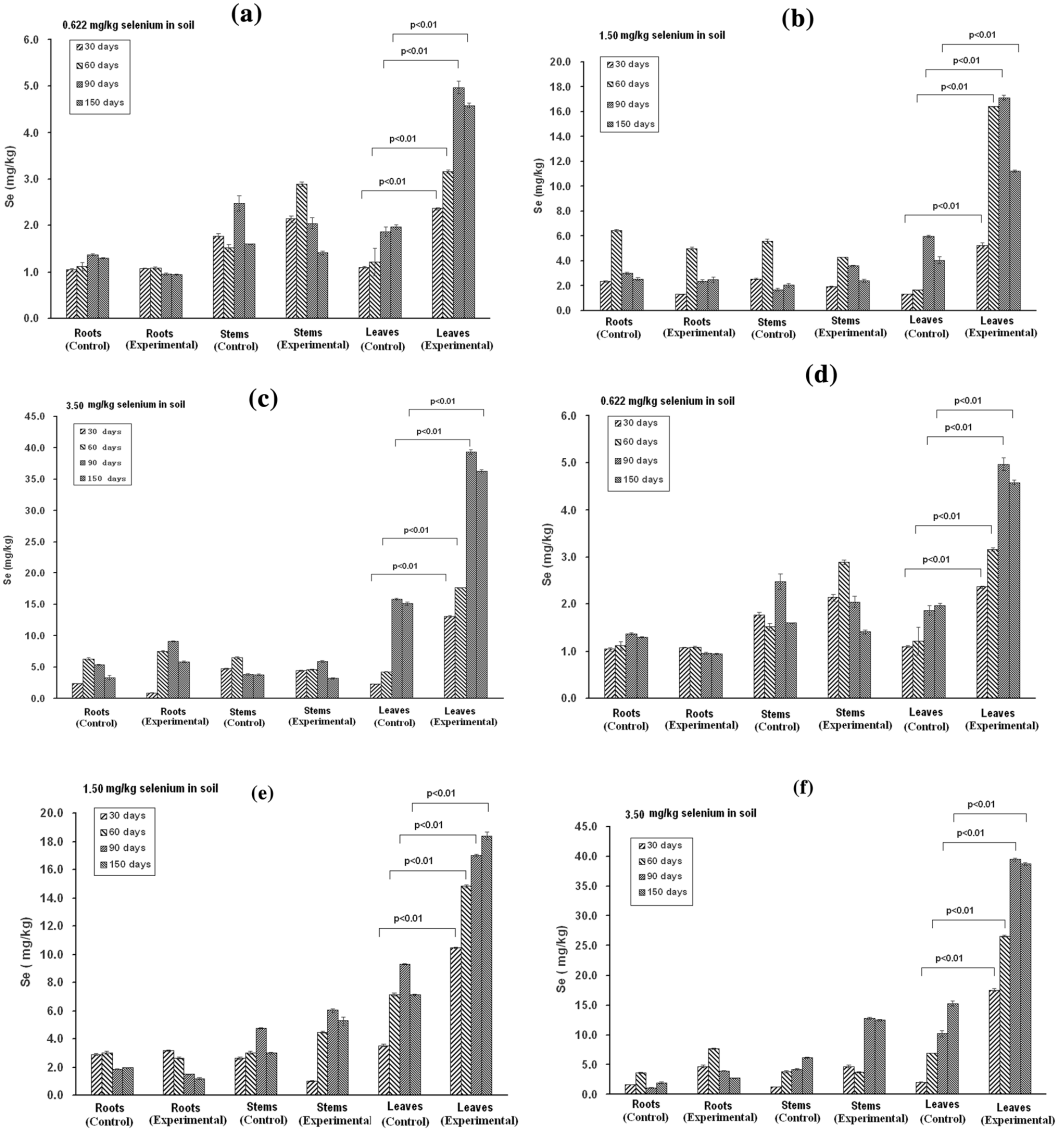
Selenium contents of roots, stems, and leaves of tea seedlings. The data were collected at 30, 60, 90, and 150 days posttreatment. **a**–**c** Bacterial inoculation by a needle injection method and **d**–**f** Bacterial inoculation by soaking tea-cutting method. **a**, **d** The data were obtained from the tea seedlings growing in soil with 0.622 mg/kg selenium; **b**, **e** The data were obtained from tea seedlings growing in soil with 1.5 mg/kg selenium; and **c**, **f** The data were obtained from tea seedlings growing in soil with 3.5 mg/kg selenium. Significant difference was marked for selenium contents of tea leaves between the control and experimental groups

In the needle injection experiment, we inoculated the bacterium by needle injection into the stems of tea seedlings growing in soil with different selenate concentrations (0.622, 1.5 and 3.5 mg/kg). After 30, 60, 90, and 180 days post injection, the roots, stems, and leaves of tea seedlings were collected, and selenium contents were measured. As shown in Fig. 6a–c, the selenium contents of the roots, stems, and leaves increased along with the increasing selenate concentration in soil, indicating that tea plants were able to become enriched for selenium to a certain extent. Nevertheless, the selenium distribution in the roots, stems, and leaves of tea plants in the control groups did not show obvious difference. Different from the control groups, all three experimental groups showed a marked selenium enrichment in tea leaves. After tea plants were inoculated with *Herbaspirillum* sp. WT00C, the selenium contents of tea leaves were approximately twice that as those for the control groups. A significant difference (*P *< 0.01) in the selenium contents of tea leaves between the control and experimental groups illustrated that the colonization of *Herbaspirillum* sp. WT00C inside tea plants strongly enhanced selenium enrichment in tea leaves. Non-selenium soil was also used to cultivate tea seedlings with or without inoculation of *Herbaspirillum* sp. WT00C, and the selenium contents in the roots, stems, and leaves of these plants was examined. In both cases, the selenium content was quite low (< 0.2 mg/kg) in the roots, stems or leaves (data not shown). Thus, selenium enrichment in tea plants depended on a selenium source in the soil, and improving selenium enrichment of tea by *Herbaspirillum* sp. WT00C only occurred when tea plants grew in those selenium-containing soils.

In the soaking tea-cutting method, we introduced the bacterium into tea seedlings by means of tea-cutting propagation. When tea cuttings were soaked in the bacterial culture, *Herbaspirillum* sp. WT00C entered tea cuttings via incisions at the lower end of each tea cutting. The tea-cutting seedlings with *Herbaspirillum* sp. WT00C were then transplanted into soils containing different concentrations of selenate as described above. Tea-cutting seedlings without bacterial inoculation were grown under the same conditions and used as a control. Figure 6d–f shows the observed selenium contents of roots, stems, and leaves of tea seedlings at different times post-transplantation. Similar to the results shown in Fig. 6a–c, the selenium contents in the roots or stems of the control and experimental groups were almost comparable. In contrast, the selenium contents in tea leaves were significantly different (*P *< 0.01). In all three treatments, the selenium contents of tea leaves in the experimental groups were more than twofold higher than that observed in the control groups. Thus, the marked selenium enrichment in tea leaves demonstrated that *Herbaspirillum* sp. WT00C had the ability to enhance selenium enrichment of tea after it colonized in tea plants.

## Discussion

A genomic survey [37] found that *Herbaspirillum* sp. WT00C, similar to *Herbaspirillum* sp. Os34 and other bacteria [43–45], possesses an intact pathway for selenocompound metabolism, where selenate is initially converted to selenite and is then further reduced to zerovalent elemental selenium or incorporated into proteins through selenocysteine. After incubation of the bacterial cells with sodium selenate, *Herbaspirillum* sp. WT00C indeed turned the medium red and depleted selenate. Furthermore, SEM, TEM, and XRD analyses confirmed that *Herbaspirillum* sp. WT00C had the ability to reduce selenate to red elemental selenium. Although a high concentration of selenate initially inhibited its growth, the bacterium recovered its ability to grow when the selenate in the medium fell to a low concentration. In view of the fact that *Herbaspirillum* sp. WT00C was able to survive in more than 200 mM selenate, this bacterium obviously had a strong capacity of selenate tolerance. Nevertheless, the detailed mechanism needs to be studied still further. In selenate reduction experiments, the cytoplasmic extract was more active than the cell-debris precipitate, suggesting that the selenate reduction primarily occurred inside bacterial cells. After selenate was reduced to elemental selenium, the elemental selenium further formed SeNPs inside bacterial cells. Those SeNPs, associated with bacterial proteins, were secreted to bacterial surfaces in the shape of selenium granules and then aggregated to form small Se-nanospheres. Small Se-nanospheres grew larger outside bacterial cells via fusion between Se-nanospheres. Finally, elemental selenium in large nanospheres further crystallized to form selenoflowers (Fig. 3). In this bacterium, selenate reduction primarily occurred inside the bacterial cell, and then Se-nanospheres grew and accumulated outside the bacterial cell, similar to reports in *Bacillus mycoides* SeITE01 [46] and *Bacillus cereus* [42] under aerobic conditions but different from those reported in *Shewanella oneidensis* MR-1 [47] *Rhodospirillum rubrum* and *E. coli* [45].

**Fig. 7:**
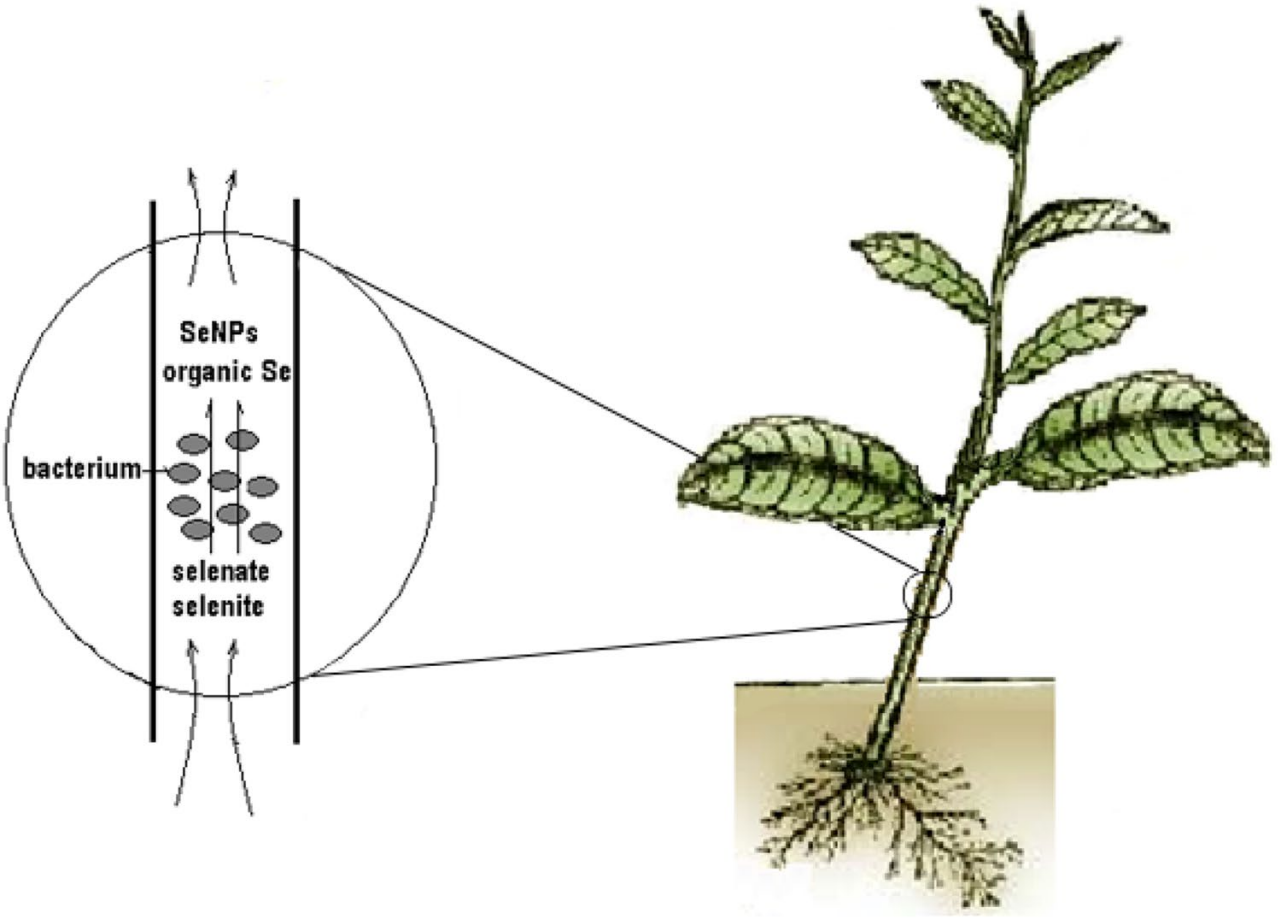
A plausible mechanism for the enhancement of selenium enrichment by *Herbaspirillum* sp. WT00C

Based on its selenium reduction and metabolic capacity, as well as endophytic host specificity, we hypothesized that *Herbaspirillum* sp. WT00C might improve selenium enrichment of tea when the bacterium colonized inside tea plants. Our results, obtained from both needle injection and soaking tea-cutting methods, demonstrated that endophytic *Herbaspirillum* sp. WT00C indeed enhanced selenium enrichment in tea leaves. Here, it is inevitable to raise a question how *Herbaspirillum* sp. WT00C improves the selenium enrichment of tea. When tea plants grow in the selenium-containing soil, selenate or selenite enters tea plants via root absorption and then reaches to the upper part of tea plants through plant vessel transportation. Inside plant cells, selenate/selenite is metabolized to selenium compounds [1, 3]. Selenate (SeO_4_^2−^) and selenite (SeO_3_^2−^), two oxidized forms (oxyanions) of selenium, usually exist in natural environments, and both compounds are soluble in water but toxic to living things [2]. Elemental selenium (Se^0^) is insoluble but exhibits no or very little toxicity [1, 13]. As it predominantly colonizes inside the stem of tea plants [31], *Herbaspirillum* sp. WT00C present in tea plant stems can reduce selenate and selenite to produce elemental selenium, selenodiglutathione, glutathioselenol, selenocysteine, selenomethionine, or selenoproteins when inorganic selenocompounds are absorbed via roots and transported into the stems of tea plants through the conducting system of tea-plant xylem. The selenate reduction of *Herbaspirillum* sp. WT00C not only decreases the toxicity of inorganic selenocompounds absorbed by plant roots to maintain vigorous growth of tea plants but also forms organic selenium and Se-nanoparticles. Organic selenocompounds can be directly accumulated and utilized by tea-plant cells, whereas atoxic Se-nanoparticles associated with proteins can be transported to tea leaves by means of plant transpiration, where Se-nanoparticles may be further metabolized to form organic selenium in tea-plant cells or be directly taken into the tissues of tea leaves (Fig. 7).

Selenium contents vary from 0.5 to 3.5 mg/kg in agricultural soil of selenium-rich areas [48]. In our study, soils containing 0.622, 1.5, and 3.5 mg/kg inorganic selenium were used for the cultivation of tea plants. When the soaking tea-cutting method was used, the selenium contents in tea leaves were 5, 18, and 40 mg/kg, respectively, two times more than those observed in the control groups. The higher selenium content observed in tea leaves demonstrates that selenium enrichment of tea is indeed improved by *Herbaspirillum* sp. WT00C. Our studies not only show the capability of *Herbaspirillum* sp. WT00C to improve selenium enrichment of tea but also offer a novel technique for field application by tea farmers.
